# Molecular Iodine/*PPARγ* Interaction in the Invasion and Angiogenesis of Neuroblastoma Xenografts

**DOI:** 10.3390/cells15131189

**Published:** 2026-06-30

**Authors:** Edgar R. Juvera-Avalos, Gustavo Orizaga-Osti, Evangelina Delgado-Gonzalez, Hilda Lomeli, Brenda Anguiano, Carmen Aceves

**Affiliations:** 1 Instituto de Neurobiología, Universidad Nacional Autónoma de Mexico, Juriquilla 76230, Queretaro, Mexico; rodrigojuvera@gmail.com (E.R.J.-A.); orizaga.osti@gmail.com (G.O.-O.); edelgado@comunidad.unam.mx (E.D.-G.); anguianoo@unam.mx (B.A.); 2 Instituto de Biotecnología, Universidad Nacional Autónoma de Mexico, Cuernavaca 62210, Morelos, Mexico; hilda.lomeli@ibt.unam.mx

**Keywords:** neuroblastoma, *MYCN*, *PPARγ*, zebrafish xenograft, molecular iodine, antioxidant, angiogenesis

## Abstract

**Highlights:**

**What are the main findings?**

- Molecular iodine (I_2_) diminishes the viability of SK-N-AS (non-*MYCN*-amplified) and SK-N-BE(2) (*MYCN*-amplified) neuroblastoma cells.
- The actions involve epigenetic (antioxidant) and genetic (activation of peroxisome proliferator-activated receptor gamma; *PPARγ*) mechanisms.

**What is the implication of the main findings?**

- The antioxidant actions impair the sustained stem state (*MYCN* and *TrkA*), whereas PPARγ activation induces gene differentiation (*FasN*, *TrkB*), thereby impairing angiogenesis and invasion capacity in vitro (wound assay) and in zebrafish xenografts.

**Abstract:**

The study investigates the impact of molecular iodine (I_2_) supplementation on the viability, invasiveness, and angiogenic potential of high-risk neuroblastoma (NB). In vitro assays were performed using NB cell lines SK-N-AS (non-*MYCN*-amplified) and SK-N-BE(2) (*MYCN*-amplified). The role of peroxisome proliferator-activated receptor gamma (*PPARγ*) was evaluated using the antagonist GW9662, gene expression (RT-qPCR), and protein levels (Western blot). In vivo, zebrafish xenografts were used to evaluate tumor size, angiogenesis, and caudal cell dissemination. I_2_ supplementation significantly decreased cell viability in both cell lines, independent of *PPARγ* activation. In SK-N-BE(2), I_2_ impaired cell migration, as measured by a wound-healing assay, in apparent independence of *PPARγ* activation. However, gene expression indicates that I_2_ acts in complex ways, including direct antioxidant effects and *PPARγ*-mediated effects. The significant decrease in reactive oxygen species levels (DCFDA staining) and the silencing of the long noncoding RNA myocardial infarction-associated transcript (*MIAT*) by I_2_ were directly associated with decreased *MYCN* and *TrkB* expression. In contrast, *PPARγ* activation was accompanied by overexpression of *FasN* and *TrkA* and a significant decrease in *Aurka*, a *MYCN*-stabilizing protein. In zebrafish, I_2_-pretreated SK-N-BE(2) xenografts exhibited a clear reduction in angiogenesis (vascular density) and a decrease in invasive capacity. In conclusion, I_2_ supplementation decreases cell viability and attenuates invasion and angiogenesis in NB cells, highlighting its potential as an adjuvant to conventional therapy for high-risk NB.

## 1. Introduction

Neuroblastoma (NB) is one of the most common extracranial solid tumors in children under five years of age. It is highly heterogeneous, with variable biological and clinical characteristics, ranging from low-risk tumors to highly invasive and chemoresistant tumors [1]. *MYCN* proto-oncogene (*MYCN*) belongs to the *MYC* gene family, which includes cellular *MYC* (*C-MYC*) and lung *MYC*. *MYCN* plays a key role in the development of neural-derived malignancies, including NB and medulloblastoma [2,3]. This gene is predominantly expressed in the peripheral neural crest during development and is vital for the proliferation, migration, and homeostasis of stem cells [4]. Low levels are associated with terminal neuronal differentiation, whereas persistent overexpression is linked to chemoresistance and invasion [5,6]. Approximately 50% of *MYCN*-amplified-NB patients have metastatic disease at the time of diagnosis, translating into poor prognosis and reduced survival [7,8,9]. *MYCN* regulates the expression of stemness-associated genes, such as octamer-binding transcription factor 4 and neurotrophic receptor tyrosine kinase 2 (*NTRK2*, *TrkB*). It also regulates angiogenesis by inducing vascular endothelial growth factor-A (*VEGFA*) [10,11]. Furthermore, researchers have proposed an association between the long noncoding RNA (lncRNA) myocardial infarction-associated transcript (*MIAT*) and *MYCN* amplification in NB [12]. *MIAT* silencing induces cell death only in cells with *MYCN* amplification, and aggressive NB exhibits *MIAT* overexpression [13]. The main trigger for *MIAT* expression is oxidative stress in the microenvironment [14]. In contrast, NB with a good prognosis is associated with the overexpression of differentiation factors, such as the neurotrophic receptor tyrosine kinase 1 (*NTRK1*, *TrkA*) and peroxisome proliferator-activated receptor gamma (*PPARγ*) [15,16]. Although *PPARγ* agonists have been shown to have clear inhibitory effects on virtually all NB cell types [17], their clinical use has been limited due to numerous adverse effects, such as hemodilution, peripheral edema, and a possible increased risk of congestive heart failure. These effects have been attributed to the ubiquitous presence of these receptors, so the search for adjuvant and organ-specific natural compounds remains of interest [18]. In this regard, molecular iodine (I_2_) is known to exert antiproliferative effects in cancer cells and synergize with the differentiating effect of all-trans retinoic acid in moderate-risk NB cell lines (SK-N-AS) and the apoptotic action of cyclophosphamide in high-risk NB cell lines (SK-N-BE(2)) [19,20]. I_2_ may exert its effects through multiple and complex mechanisms [21]. Antioxidant actions have been described, including the direct neutralization of reactive oxygen species (ROS) [22] and the release/activation of nuclear erythroid factor 2 (*Nrf2*), a master regulator of antioxidant genes [23]. I_2_ can also exert epigenetic effects by modulating methylases [24] and, ultimately, by regulating gene expression via activation of *PPARγ* [21]. In the latter, I_2_ reacts with arachidonic acid to form the lipid 6-iodolactone, a specific ligand for *PPARγ* receptors [25]. Arachidonic acid concentrations in cancer cells are approximately ten times higher than in non-cancer cells, making I_2_ a suitable candidate for therapeutic application in specific organs [26]. This work analyzes the effects of I_2_ supplementation on the viability, angiogenesis, and invasiveness of high-risk NB using in vitro and zebrafish xenograft models [27] and focuses on the role of *PPARγ* in these effects.

## 2. Materials and Methods

### 2.1. Reagents

Sublimated iodine was purchased from Macron-Avantor (Center Valley, PA, USA), and its concentration was verified by titration with sodium thiosulfate. GW9662 (GW), a PPARγ-specific antagonist, was purchased from Sigma-Aldrich (St. Louis, MO, USA), and Rosiglitazone (RGZ), an agonist, from Cayman Chemicals (Los Angeles, CA, USA). Fast DiI™ oil red dye (DiI) (Cat. No. D3899) was obtained from Thermo-Fisher Scientific (Waltman, MA, USA). N-phenylthiourea (PTU) (Cat. No. P7629-10G) and ethyl 3-aminobenzoate methanesulfonate salt (Cat. No. A5040-25G) were obtained from Sigma-Aldrich (St. Louis, MO, USA). H2DCFDA (HY-D0940) was obtained from MedChemExpress (Monmouth Junction, NJ, USA). Hydrogen peroxide (Cat. No 56001) was obtained from Fermont (Monterrey, NL, Mexico).

### 2.2. Cell Culture

Human NB cell lines SK-N-AS (CRL-2137) and SK-N-BE(2) (CRL-2271) were purchased from the American Type Culture Collection (Manassas, VA, USA). The media were supplemented with fetal bovine serum (FBS, 10%) and penicillin–streptomycin (2%) from Invitrogen (Carlsbad, CA, USA) in a humidified chamber with a 5% CO_2_ atmosphere and 95% air at 37 °C. For zebrafish experiments, the SK-N-BE(2) cells were labeled with 4 µg/mL DiI one day before the experiments were performed, following the manufacturer’s instructions.

### 2.3. Cell Viability

A total of 50,000 cells/well were seeded on 24-well plates. After 24 h, 400 µM I_2_ was added for 96 h. In the GW/I_2_ (0.5 µM) and GW/RGZ (0.1 µM) groups, GW was administered 2 h before I_2_ or RGZ treatment. After treatment, viability was measured using a trypan blue exclusion test on a hemocytometer and light microscopy; results were reported as a fold change relative to the control. All experiments were carried out in triplicate.

### 2.4. Gene Expression

*PPARγ*, *MYCN*, *MIAT*, *VEGFA*, *TrkA*, *TrkB*, *N-cadherin*, *Vimentin*, Aurora kinase A (*Aurka*), fatty acid synthase (*FasN*), and *β-actin* were analyzed by RT-qPCR in SK-N-BE(2) cells. Briefly, total RNA was isolated using Trizol reagent (Life Technologies, Inc., Carlsbad, CA, USA). RNA (2 µg) was reverse transcribed (RT) using oligo-deoxythymidine (Invitrogen, Waltham, MA, USA). Real-time PCR was performed on the Rotor-Gene 3000 sequence detector system (Corbett Research, Mortlake, NSW, Australia) using SYBR Green as a DNA amplification marker (gene-specific primers are listed in Table 1). Relative mRNA levels were normalized to the mRNA level of *β-actin*.

### 2.5. Antioxidant Assay

A total of 5000 cells per well were seeded into 96-well plates. GW was administered 2 h before supplementation with 400 µM I_2_ in the respective groups, and cells were cultured for 96 h. ROS levels were quantified using H2DCFDA (DCFDA) staining according to the manufacturer’s protocol. DCFDA (5 µM) was added to the culture plate and incubated for 30 min at 37 °C in the dark. Hydrogen peroxide (100 µM) was used as a positive control. Fluorescence intensity was measured using a Varioskan LUX Multimode Microplate Reader (Thermo-Fisher Scientific, Waltman, MA, USA). Data were expressed as maximum DCFDA fluorescence levels relative to control cells.

### 2.6. Wound Healing Assay

A total of 100,000 cells were seeded into 12-well plates and cultured under standard conditions until they reached 80% confluence. A wound was then created in each well via a 200 µL pipette tip, and the wells were washed with phosphate-buffered saline to remove any detached cells. The medium was replaced with fresh medium supplemented with 5% FBS, and the corresponding treatment was added to each well. Photographs were taken at 0 and 24 h post-wounding. The open wound area was analyzed using ImageJ 1.54 (NIH, Bethesda, MD, USA).

### 2.7. Western Blot

Western blot analysis of MYCN and PPARγ proteins in NB cell lines was performed using chemiluminescence. Briefly, 25 μg of protein per lane were separated by electrophoresis in 12% acrylamide gel, and proteins were later transferred to a nitrocellulose membrane (Bio-Rad, Hercules, CA, USA). The unspecified reaction was blocked for 2 h with Tris-buffered saline with Tween-20 containing 5% skimmed milk powder. The membranes were treated with polyclonal antibodies against MYCN (B8.4.B) (sc-53993, Santa Cruz Biotechnology, Los Angeles, CA, USA) and PPARγ (SAB5700625; Sigma-Aldrich, St. Louis, MO, USA). As a secondary antibody, goat anti-mouse-HRP (62-6520) and goat anti-rabbit-HRP (65-6120) (Invitrogen, Waltham, MA, USA) were used. Proteins were visualized using chemiluminescent detection with the Clarity Western ECL substrate (Bio-Rad). The blots were visualized and imaged using Image Lab^TM^ (Bio-Rad).

### 2.8. Zebrafish Xenograft Model

Wild-type (WT) zebrafish embryos were maintained at 28 °C in E3 embryo medium containing, per liter: 0.286 g NaCl, 0.0126 g KCl, 0.048 g CaCl_2_·2H_2_O, and 0.081 g MgSO_4_·7H_2_O (pH 7.2), supplemented with 0.2 mM PTU. Cells were labeled as previously described [28]. DiI-labeled SK-N-BE(2) cells were injected into the perivitelline space of two-day-old zebrafish larvae. Larvae were incubated at 28 °C in E3/PTU medium, and cancer cell dissemination to the caudal vein plexus was analyzed after three days. Larvae were photographed under a Nikon (Tokyo, Japan) light/fluorescence microscope (10×/0.30 magnification). For the tumor angiogenesis experiment, DiI-labeled cells were injected into the perivitelline space of two-day-old transgenic Tg(Fli1:EFGP)^y1^ zebrafish larvae expressing the green EGFP protein in vascular endothelial cells with 80% ECM gel as previously described [28]. Larvae were incubated at 28 °C in E3/PTU medium for 72 h, fixed overnight in 4% paraformaldehyde, and mounted with an antifade reagent containing DAPI. All experiments were performed using larvae obtained from at least three independent zebrafish clutches. Z-stack images of primary tumors and intratumoral vessels were acquired using an upright confocal microscope from THOR LABS (Newton, NJ, USA). The percentage of intratumoral angiogenesis was quantified using ImageJ 1.54 (NIH, Bethesda, MD, USA).

## 3. Results

### 3.1. Participation of PPARγ in Cell Viability 

**Figure 1:**
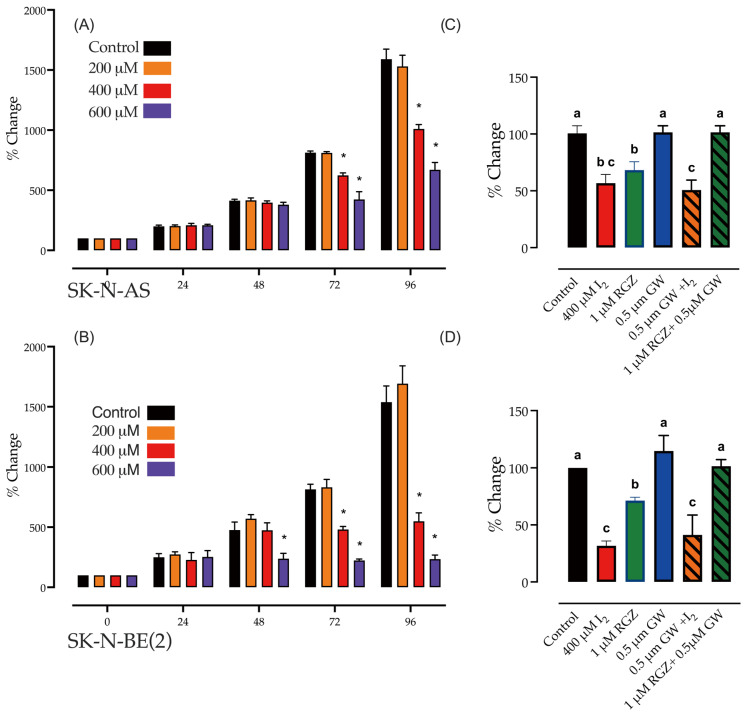
Response to I_2_ and the participation of *PPARγ* in cell viability. (**A**,**B**) The trypan blue exclusion test was used to assess the viability of SK-N-AS and SK-N-BE(2) cells at different I_2_ concentrations. (**C**,**D**), response of viability in the presence of I_2_ (400 µM) and *PPARγ* agonist (RGZ 1 µM) and antagonist (GW; 0.5 µM). All experiments were carried out for 96 h. Data are representative of at least three independent experiments per duplicate and expressed as the mean ± SD. The data were analyzed via one-way ANOVA. Asterisks and letters denote statistical differences (*p* < 0.05).

Figure 1 summarizes the viability of NB cells at several concentrations of I_2_ supplementation and the involvement of *PPARγ*. Both cell lines are sensitive to I_2_, and although the IC50 values are similar (380 and 368 µM), the SK-N-BE(2) cell line is more sensitive, showing inhibition within 48 h. *PPARγ* participation was analyzed using the agonist RGZ and the antagonist GW. Both cell lines exhibited decreased viability upon *PPARγ* activation with RGZ, and the presence of GW reversed this effect. Conversely, although I_2_ supplementation exerted a similar decrease, GW did not cancel or reverse it, suggesting that this inhibition is independent of *PPARγ*.

### 3.2. Antioxidant Effect and Molecular Response

**Figure 2:**
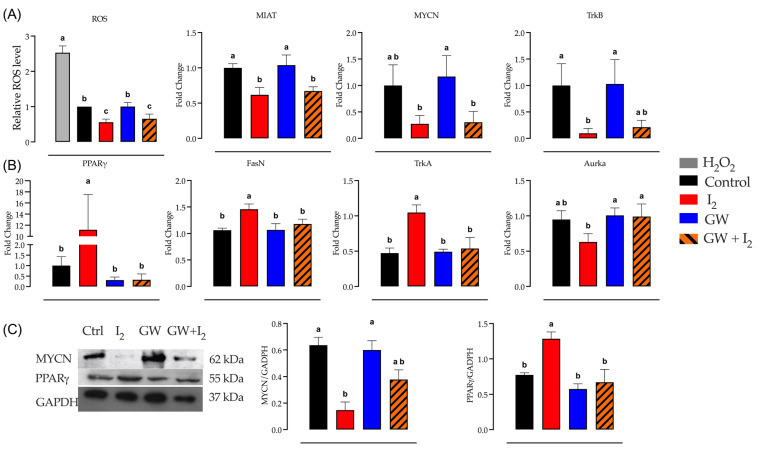
Antioxidant effect and molecular responses to I_2_ and GW in SK-N-BE(2) cells. (**A**) ROS levels were analyzed by the DCFDA assay using 100 μM hydrogen peroxide (H_2_O_2_) as a positive control, and the expression of genes responds independently of *PPARγ* activation. (**B)** Gene expression depends on *PPARγ* activation and was normalized to β-actin (qPCR). (**C**) Protein content (Western blot) of MYCN and *PPARγ*. Protein content was normalized by glyceraldehyde-3-phosphate dehydrogenase (GAPDH). All treatments included I_2_ (400 μM), PPARγ antagonist GW9662 (0.5 μM; GW), and the combination (GW + I_2_) for 96 h. Data are representative of at least three independent experiments per duplicate and are expressed as the mean ± SD. The data were analyzed via one-way ANOVA. Letters denote statistical differences (*p* < 0.05).

To analyze *PPARγ’s* role in molecular responses to I_2_, genes associated with oxidative status or directly regulated by *PPARγ* were examined. Figure 2 summarizes the antioxidant effect and the expression of different messengers in response to the I_2_ supplementation alone or in the presence of the *PPARγ* antagonist GW. Panel A shows lower ROS levels and inhibition of gene expression associated with oxidative stress (*MIAT*), proliferation, and chemoresistance (*MYCN* and *TrkB*) in the I_2_-supplemented groups (I_2_ and GW + I_2_). GW did not counteract these responses. Panel B shows overexpression of genes related to differentiation, such as *PPARγ*, *FasN*, and *TrkA*, and decreased expression of *Aurka*, which encodes Aurora kinase A and is involved in *MYCN* stabilization. The presence of GW abolished all these responses. Panel C corroborates that I_2_ increases PPARγ protein levels and diminishes MYCN (Western blot).

### 3.3. Invasion Responses

**Figure 3:**
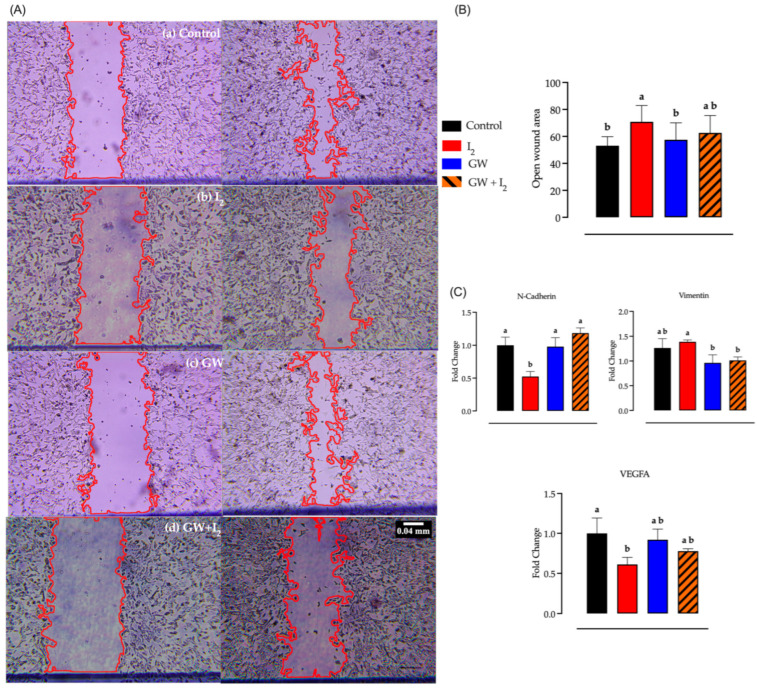
Invasive response of the SK-N-BE(2) cells to I_2_. (**A**) Wound healing assay; (**a**–**d**) representative micrographs of each condition (10× magnification) of the open wound and quantification of open wound area after 24 h post-treatment. The wound area is highlighted in red along the borders. (**B**) Quantification of the open wound area after treatment. (**C**) Genes related to invasiveness. Expression was normalized to ß-actin (quantitative PCR). Data are representative of three independent experiments and expressed as the mean ± SD. The data were analyzed via one-way ANOVA and Tukey’s test. Letters denote statistical differences (*p* < 0.05).

To assess the effect of I_2_ on migration in vitro, we performed a wound healing assay. Wound healing was measured at 24 h post-treatment (Figure 3B), revealing that I_2_ reduces cell migration. This effect appears to be partially mediated by *PPARγ*, since the presence of GW + I_2_ does not completely inhibit wound closure. Figure 3B shows the inhibitory effect of I_2_ on genes associated with invasion, such as *N-cadherin* and *VEGFA*.

### 3.4. In Vivo Responses

**Figure 4:**
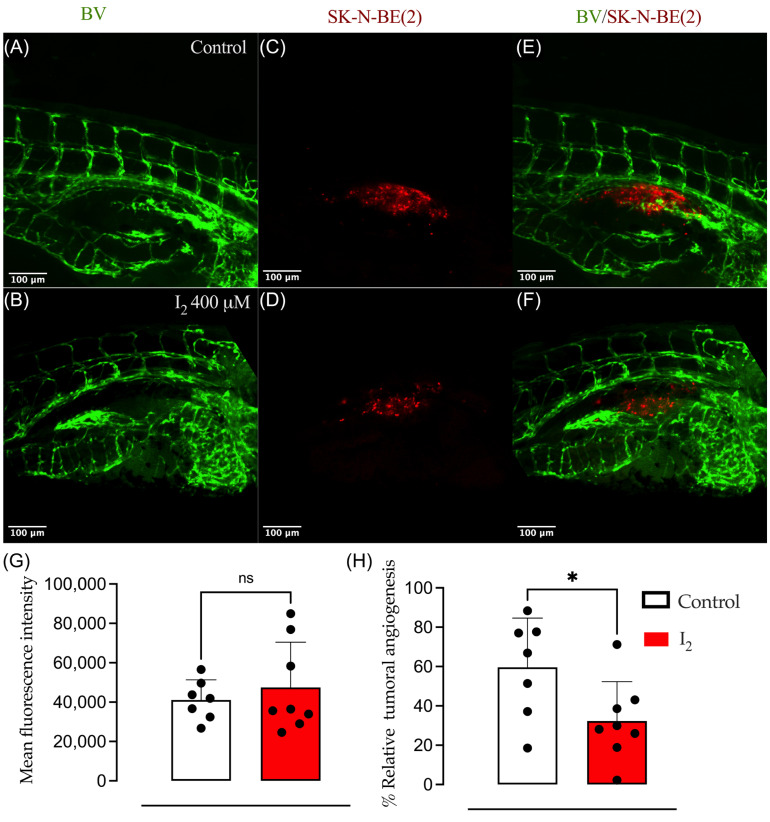
Xenograft density and angiogenesis in zebrafish. Representative micrographs of xenograft size (red) and intratumoral blood vessels (green) (BVs) in zebrafish larvae. Standard (control) and 400 μM I_2_-pre-treated SK-N-BE(2) cells were incubated for 96 h. Between 400 and 600 DiI-labeled cells with extracellular matrix were subcutaneously implanted into two-day-old Tg (Fli1:EFGP)^y1^ zebrafish embryos. Xenograft density and intratumoral BVs were analyzed by confocal microscopy three days after injection. Each dot represents an independent larva. (**A**,**B**) BVs in zebrafish larvae. (**C**,**D**) Xenograft density (red). (**E**,**F**) Intratumoral BV densitometry (angiogenesis). Scale bar = 100 µm. (**G**) Mean fluorescence intensity among subjects. (**H**) Percentage of vascular area in the tumor (relative tumoral angiogenesis). Data are expressed as the mean ± SD and were analyzed using Student’s *t*-test; * *p* < 0.05.

To assess the tumor implantation and angiogenic capacity of normal or I_2_-pretreated SK-N-BE(2) cells, xenografts were generated in Tg (Fli1:EFGP)^y1^ zebrafish larvae at 48 h postfertilization. The larvae exhibited green, fluorescent blood vessels. Cells were grown under standard conditions or supplemented with 400 µM I_2_ for 96 h. Between four hundred and six hundred DiI-label cells (red) were injected into each larva. The results showed that both populations formed xenografts with similar growth (as measured by densitometry), but the I_2_-pretreated cells exhibited a significant decrease in tumoral angiogenesis (Figure 4).

**Figure 5:**
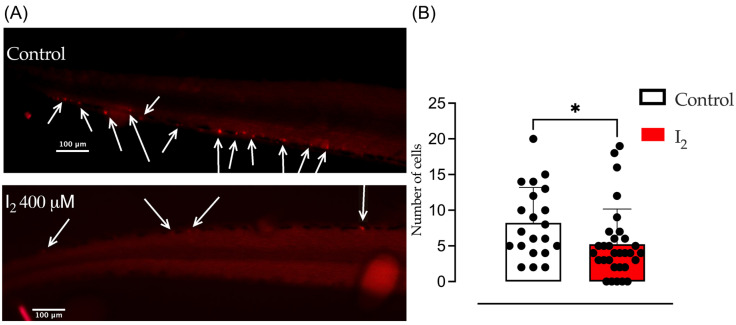
Caudal migration of SK-N-BE(2) cells in zebrafish. Representative micrograph of the caudal region and quantification of control (*n* = 21) and I_2_-pretreated (400 μM) SK-N-BE(2) cells (*n* = 31). (**A**) Between 400 and 600 DiI-label cells were subcutaneously implanted into wild-type zebrafish embryos, white arrows indicate NB cells in zebrafish. (**B**) Quantification of caudal cells was analyzed after 96 h. Each dot represents an independent larva. Data are expressed as the mean ± SD and were analyzed using Student’s *t*-test; * *p* < 0.05.

To analyze invasion capacity in vivo, standard and I_2_-pretreated SK-N-BE(2) cells were subcutaneously implanted into two-day-old wild-type zebrafish embryos. In Figure 5, a distinct presence of red DiI-labeled cells is evident in the caudal region in both groups (fluorescence microscopy). The results corroborated the invasive capacity of these NB cells, and the quantification shows a significant decrease in cell number in the I_2_-pretreated group.

## 4. Discussion

Studies suggest that *PPARγ* agonists can promote differentiation and inhibit NB cell growth, highlighting their therapeutic potential [16]. All NB cell lines express *PPARγ*, with higher expression levels observed in differentiated cells, suggesting an active role for *PPARγ* in NB cell differentiation [17]. In a 2004 study, Servidei and colleagues evaluated two *PPARγ* agonists (15-deoxy-PGJ2 and RGZ) in eight NB cell lines with different phenotypes, including N-type (neuroblastic) and S-type (stromal) cells. Both ligands inhibited cell growth across all lines, with N-type cells exhibiting greater susceptibility, likely due to increased sensitivity to apoptosis [29]. Our results revealed that I_2_ reduced the viability of both SK-N-AS (stromal) and SK-N-BE(2) (neuroblastic) cells, confirming the high sensitivity of N-type cells.

*MYCN* overexpression has been linked to a more aggressive NB and poor prognosis [2,30]. Previous work from our group demonstrated that I_2_ reduces *MYCN* expression and increases *PPARγ* expression in NB xenografts in mice [20]. These findings align with our in vitro results, where I_2_ downregulated *MYCN* and upregulated *PPARγ*. To explore the roles of *PPARγ* in the effects of I_2_, we used the *PPARγ* antagonist GW. Interestingly, I_2_ consistently downregulated *MYCN* and *TrkB*, genes associated with aggressive NB, even after *PPARγ* inhibition with GW, suggesting that I_2_ partially acts through a *PPARγ*-independent pathway.

To elucidate the possible mechanism by which I_2_ negatively regulates *MYCN*, we evaluated its antioxidant effect. DCFDA analyses show that I_2_-supplemented groups exhibit lower ROS levels, and GW does not modify this effect. These results agree with previous reports showing that with micromolar iodine concentrations, there is a decrease in ROS-induced damage in rabbits and humans [31,32] and a prevention of lipid peroxidation in various normal and cancerous vertebrate tissues [21,33]. Physiological levels of ROS regulate signal transduction, gene expression, and proliferation. However, shifts from physiological to pathophysiological levels are known as oxidative stress. Oxidative stress damages lipids, proteins, and nuclear and mitochondrial DNA and is involved in epigenetic modifications that contribute to carcinogenesis. Epigenetic modifications include methylases, demethylases, and non-coding RNAs [34]. *MIAT* is an lncRNA originally identified in patients with myocardial infarction and is overexpressed in response to oxidative stress across various normal and cancerous tissues, including NB [14]. Aggressive NB exhibits *MIAT* overexpression, and its silencing induces cell death only in cells with *MYCN* amplification [13]. In this work, we demonstrate that I_2_ supplementation significantly reduced both ROS levels and *MIAT* expression, which are associated with the decreases in *MYCN* and *TrkB* expression. As we mentioned before, *MYCN* is a master gene vital for stem cell proliferation, migration, and homeostasis that, under normal conditions, decreases during terminal neuronal differentiation, but its persistent overexpression is linked to chemoresistance and invasion [6]. Moreover, *MYCN* directly regulates *TrkB* overexpression, which is associated with a poor prognosis and chemoresistance [3].

Regarding genes associated with *PPARγ*, our results show that I_2_ supplementation significantly increased *PPARγ* RNA and protein levels, as well as *FasN* and *TrkA* expression, indicating that I_2_ promotes cell differentiation [15]. Fatty acid synthase is an enzyme encoded by the *FasN* gene. Its main function is to catalyze fatty acid synthesis, and although it can act as a protumoral agent in some contexts, it can also participate in the differentiation process [35]. Since *PPARγ* directly regulates *FasN* expression, this corroborates the idea that I_2_ activates these receptors [36].

With respect to *Trks*, both *TrkA* and *TrkB* are part of the receptor tyrosine kinase family and are critically involved in the regulation of neuronal development [3]. *TrkA* expression is significantly lower in aggressive NB, and it is inversely associated with *MYCN* amplification [37]. Elevated *TrkA* expression has been reported in patients with spontaneously regressed stage IV-S NB and in those with well-differentiated NB [38,39]. Furthermore, *TrkA* interacted with *PPARγ* during PC12 cell differentiation, leading to mutual activation [40].

Another gene of interest was *Aurka*. *Aurka* encodes Aurora serine-threonine kinase A, which is vital in centrosome maturation and segregation, spindle assembly, and cell cycle regulation [41]. The importance of *Aurka* as a therapeutic target in NB stems from its dual functions: catalytic activities during mitosis and kinase-independent functions, particularly *MYCN* protein stabilization [42]. Elevated *Aurka* expression has been associated with poor overall survival, and *Aurka*-targeted therapy inhibited cell growth, reduced *MYC* protein levels, and inhibited tumor growth in a murine xenograft model [43]. Moreover, *Aurka* expression is dependent on *PPARγ* activation [44]. Our results show that I_2_ supplementation decreases its expression and corroborate the dependence on *PPARγ* (canceled by GW). In addition, the very low MYCN protein content (Western blot) observed in our I_2_ samples was consistent with *Aurka*’s crucial function in MYCN protein stability [42].

Analysis of the invasive capacity of SK-N-BE(2) cells in vitro using the wound healing assay revealed that I_2_-pretreated cells exhibit reduced motility. This effect appears to be partially mediated by *PPARγ* activation. Interestingly, this partial regulation is corroborated by the complete involvement of *PPARγ* in *N-Cadherin* expression, whereas *VEGFA* expression appears to be only partially dependent on this pathway. Cadherins constitute a large superfamily of adhesion molecules comprising more than 80 members, among which epithelial (E) and neuronal (N) cadherins are the most extensively studied. *E-Cadherin* is predominantly expressed in epithelial cells, promoting cell–cell adhesion, whereas *N-Cadherin* is mainly expressed in neuronal tissues and fibroblasts. *N-Cadherin* plays a critical role in neural crest cell migration during early embryonic development. In neural malignancies, metastasis progression is often associated with the loss of *E-Cadherin* and the de novo expression of *N-Cadherin*, a phenomenon known as the “cadherin switch,” which contributes to the establishment of epithelial–mesenchymal transition (EMT) [45]. Although the role of *PPARγ* in *N-Cadherin* expression in NB remains largely unexplored, *PPARγ*-mediated inhibition of *TGF-β1*-induced EMT has been reported in several cancer types [46]. Regarding *VEGFA*, our findings are consistent with the well-established direct involvement of *MYCN* in *VEGFA* overexpression in both normal and tumor tissues [47].

Finally, using the zebrafish xenograft model, we evaluated the angiogenic and invasive potential (caudal migration) of the I_2_-preselected cells in vivo relative to control cells. Both groups displayed comparable implantation rates; however, I_2_-pretreated cells exhibited a reduced capacity to induce intratumoral angiogenesis (50%) and decreased caudal migration (38%), indicative of lower invasive potential. We are aware that a potential temperature-related limitation of the zebrafish xenograft model is the temperature mismatch between the fish host and mammalian tumor cells, as human cells are maintained at 28 °C rather than their physiological temperature. Nevertheless, the zebrafish model is widely accepted and validated for studies of metastasis and cancer pharmacology [48]. Mammalian cancer cells typically display optimal proliferative activity at 37 °C, and at lower temperatures (e.g., 28 °C), they decrease their proliferative capacity, although their angiogenic and metastatic/invasive potential does not appear to be significantly altered [49,50]. On the other hand, the optimum temperature for fish development is around 26–28 °C. Increasing the temperature above 30 °C causes stress in the organisms, accompanied by metabolic dysfunctions and significant malformations [51]. To address this limitation, many studies prioritize maintaining physiological conditions for the host organism while assessing tumor-cell behaviors, such as angiogenesis and short-term invasion (48–72 h) [52]. In our study, we used a temperature of 28 °C to prioritize fish welfare. Therefore, the reduced angiogenic and invasion capacities observed in I_2_-pretreated cells are more likely attributable to the treatment itself than to differences in the host microenvironment. Furthermore, the concordant inhibitory effects observed in both the in vitro wound assay (37 °C) and the caudal migration (28 °C) support the notion that the anti-invasive effects of I_2_ are maintained across these temperature conditions.

## 5. Conclusions

Molecular iodine induces gene remodeling that decreases the proliferative and invasive potential of moderate- and high-risk NB cells through direct antioxidant effects and *PPARγ*-dependent pathways. This consistent beneficial effect supports the use of I_2_ supplementation as an adjuvant to conventional NB therapies.

## Figures and Tables

**Table 1:** Oligonucleotide sequences.

Target	Reference	Sense	Antisense
*PPARγ*	NM_001354666.3	CGACATTCAATTGCCATGAG	GACCACTCCCACTCCTTTGA
*MYCN*	XM_054342160.1	ACCCTGAGCGATTCAGATGAT	GTGGTGACAGCCTTGGTGTT
*VEGFA*	NM_001025366.3	GACACACCCACCCACATACA	ACATCCTCCTCCCAACTCAA
*TrkA*	NM_001012331.2	CATCGTGAAGAGTGGTCTCCG	GAGAGAGACTCCAGAGCGTTGAA
*TrkB*	XM_054363028.1	TCGTGGCATTTCCGAGATTGG	TCGTCAGTTTGTTTCGGGTAAA
*N-cadherin*	NM_001317185.2	CAGTGGCCACCTACAAAG	AAATGAAACCGGGCTATC
*Vimentin*	NM_003380.5	GAGAACTTTGCCGTTGAAGC	GCTTCCTGTAGGTGGCAATC
*Aurka*	NM_001323304.2	ACCTGTTAAGGCTACAGCTCCA	AAGGACACAAGACCCGCTGA
*β-actin*	NM_001101.5	CCATCATGAAGTGTGACGTTG	ACAGAGTACTTGCGCTCAGGA
*MIAT*	NR_185983.1	GCTCACACCTCCTATTCCT	CTTCACCAACTCTCCCACT
*FasN*	NM_004104.5	ATGCTGAACGACATCGCGG	GAATCTCGGAAGCGGTCCAG

## Data Availability

The data presented in this study are available at Supplementary Materials and upon specific request from the corresponding author.

## Supplementary Materials

The following supporting information can be downloaded at: https://www.mdpi.com/article/10.3390/cells15131189/s1.

## Abbreviations

NB	Neuroblastoma
*MYCN*	MYCN proto-oncogene, bHLH transcription factor
*PPARγ*	Peroxisome proliferator-activated receptor gamma
*VEGFA*	Vascular endothelial growth factor A
*TrkA*	Neurotrophic receptor tyrosine kinase 1
*TrkB*	Neurotrophic receptor tyrosine kinase 2
*N*-*cadherin*	Neural cadherin (CDH2)
*Aurka*	Aurora kinase A
*MIAT*	Myocardial infarction-associated transcript
*FasN*	Fatty acid synthase
DCFDA	2′,7′-Dichlorofluorescein
PTU	N-phenylthiourea
WT	Wild Type
GW	GW9662
RGZ	Rosiglitazone
DiI	Fast DiI™ oil red dye
