# Supplementary material for: Molecular Iodine/PPARγ Interaction in the Invasion and Angiogenesis of Neuroblastoma Xenografts
Source: Cells. 2026 Jun 30;15(13):1189. doi: 10.3390/cells15131189 (PMC13359963; doi:10.3390/cells15131189)
Supplement: Supplementary file 1 [file cells-15-01189-s001.zip › cells-4248382-supplementary.pdf]

Raw Data

Figure S1. Response to the molecular iodine and the participation of PPARγ in cell

viability 1 A. SKNAS

Table format: XY	X	Group A				Group B				Group C				Group D		
	Horas	Control				200 µM				400 µM				600 µM		
	X	1	2	3	4	1	2	3	4	1	2	3	4	1	2	3
1	0	100.00	100.00	100.00		100.00	100.00	100.00		100.00	100.00	100.00		100.00	100.00	100.00
2	24	200.00	210.00	190.00		212.00	200.00	195.00		220.00	215.00	190.00		200.00	213.00	214.00
3	48	420.00	400.00	421.00		400.00	438.00	410.00		400.00	380.00	410.00		400.00	380.00	360.00
4	72	800.00	812.00	827.00		812.00	820.00	800.00		600.00	640.00	630.00		350.00	450.00	470.00
5	96	1500.00	1603.00	1667.00		1634.00	1456.00	1500.00		1000.00	980.00	1050.00		600.00	710.00	700.00

1 C.

Group A	Group B	Group C	Group D	Group E	Group F
Control	400 µM I_2	1 µM RZG	1 µM RGZ+ 0.5µM GW	0.5 µm GW9662	0.5 µm GW9662 +I_2
100.0	67.00	72.0	98	98.0	48
98.0	58.00	58.0	110	110.0	60
94.0	52.00	68.0	100	100.0	55
110.0	50.00	75.0	98	98.0	40

SK-N-BE(2)

1 B

Table format: XY	X	Group A				Group B				Group C				Group D		
	Horas	Control				200 µM				400 µM				600 µM		
	X	1	2	3	4	1	2	3	4	1	2	3	4	1	2	3
1	0	100.00	100.00	100.00		100.00	100.00	100.00		100.00	100.00	100.00		100.00	100.00	100.00
2	24	243.36	222.38	282.52		268.39	296.65	254.27		296.50	205.59	181.82		310.98	236.43	209.09
3	48	462.94	548.25	418.18		607.97	561.33	542.03		406.99	528.67	488.11		185.91	261.52	264.80
4	72	808.39	860.14	774.83		817.13	903.15	774.13		471.33	509.09	462.94		236.61	214.82	217.51
5	96	1574.90	1650.90	1389.80		1732.40	1816.00	1528.80		520.47	628.49	496.88		194.36	252.18	255.34

1 D

Group A	Group B	Group C	Group D	Group E	Group F
Control	400 µM I_2	1 µM RZG	1 µM RGZ+ 0.5µM GW	0.5 µm GW9662	0.5 µm GW9662 +I_2
100.000000	31.630000	72.0	98.0	129.279580	58.571429
100.000000	37.618300	70.0	110.0	123.076923	23.076923
100.000000	28.571429	68.0	100.0	103.571429	53.571429
100.000000	28.888889	75.0	98.0	103.333333	30.000000

Figure S2 Antioxidant effect and molecular responses to I_2 and GW in SK-N-BE(2) cells

2 A) ROS

	Group A	Group B	Group C	Group D	Group E
	Control P 100 uM H_20_2	Control	I_2	GW	GW+I_2
1	4.808	1.835	0.9846	1.60300	0.9610
2	4.255	1.724	0.8743	1.63400	0.9642
3	4.066	1.630	0.7506	1.62327	1.1080
4	3.978	1.569	0.8680	1.93200	0.9132
5	4.203	1.567	0.9391	1.57300	1.0040
6	4.340	1.492	0.7102	1.56980	1.1450
7	3.677	1.501	0.8231	1.62580	1.0760
8	3.531	1.571	0.8986	1.35200	0.8087
9	3.361	1.429	1.0920	1.39856	1.3190

MIAT

Group A	Group B	Group C	Group D
CTRL	I_2	GW9662	GW+I_2
1.000596723	0.499067520	1.148264168	0.614553955
0.941609810	0.689567851	1.091577118	0.735892474
1.057793467	0.665110039	0.875160380	0.662342441

MYCN

Group A	Group B	Group C	Group D
control	I_2	GW9662	GW+I_2
0.606517	0.156293	1.456466	0.169290
1.384253	0.453144	1.329862	0.205337
1.009230	0.217562	0.722058	0.540239

TrkB

Group A	Group B	Group C	Group D
CTRL	I_2	GW9662	GW+I_2
0.559111030	0.200098680	0.524426500	0.359062550
1.371929840	0.063254290	1.145589170	0.109371370
1.068959140	0.027936060	1.417950410	0.169732000

2 B)

PPARY

Group A	Group B	Group C	Group D
control	I_2	GW9662	GW+I_2
0.535093784	6.906063862	0.302574786	0.558784410
1.094569586	8.200691804	0.165552841	0.019713991
1.370336630	18.447631250	0.457030919	0.400635919

FasN

Group A	Group B	Group C	Group D
CTRL	I_2	GW9662	GW+I_2
1.096677680	1.550064347	1.020000000	1.182257692
1.068000000	1.469649410	0.980000000	1.267537736
1.017099898	1.350472204	1.200000000	1.088000000

TrkA

Group A	Group B	Group C	Group D
CTRL	I_2	GW9662	GW+I_2
0.410000000	1.009841220	0.458225125	0.650277978
0.460000000	0.963900000	0.531863697	0.359516147
0.550000000	1.168071407	0.486033773	0.604631678

Aurka

Group A	Group B	Group C	Group D
CTRL	I_2	GW9662	GW+I_2
1.092330000	0.689968774	1.128342477	0.803250990
0.882565897	0.704901160	0.938085717	1.157658829
0.872101681	0.497421772	0.953432304	1.011293957

2 C) Westernblot

MYCN

Group A	Group B	Group C	Group D
ctrl	I_2	GW	GW+I_2
0.75280318	0.24322900	0.73776599	0.48467585
0.55185427	0.03126890	0.51263000	0.23903157
0.60292167	0.16564211	0.54802142	0.41141922

PPARγ

Group A	Group B	Group C	Group D
ctrl	I_2	GW	GW+I_2
0.801673743	1.47806447	0.70284000	1.01302462
0.713023370	1.18638631	0.57474455	0.60651985
0.806438940	1.19162811	0.44889668	0.39514587

Figure S3 Wound Healing

Assay 3 B) Open wound area

Group A	Group B	Group C	Group D
Control	I2	gw	gw+i2
54.28571430	55.88235290	54.27654000	58.68470000
50.00000000	71.14660000	31.42857140	47.05882350
53.65853660	69.74520000	54.05405410	58.49056600
60.22727270	93.10344830	64.70588240	80.00000000
42.85714290	85.29411760	67.56756760	72.72727270
51.59399000	87.17948720	71.42857140	76.59574470
46.80851060	68.54673000	43.24324320	50.00000000
47.72727270	58.82352940	52.38095240	41.37931030
48.78048780	63.41463410	47.61904760	52.77777780
55.25780000	59.45945950	69.23076920	69.76744190
60.00000000	62.16216220	66.66666670	70.45454550
66.66666670	75.00000000	68.18181820	73.46938780

3 C

N-cadherin

Group A	Group B	Group C	Group D
CTRL	I_2	GW9662	GW+I_2
0.911127266	0.533863135	0.857291801	1.146143109
1.141338788	0.444261871	1.128301364	1.133717373
0.947533947	0.595209048	0.951895344	1.274285295

Vimentin

Group A	Group B	Group C	Group D	G
control	I_2	GW9662	GW+I_2	
1.049533029	1.343794005	0.800929725	1.075306226	
1.329000000	1.406600000	1.124861648	0.936108440	
1.409074185	1.412500000	0.962000000	1.022943808	

VEGFA

Group A	Group B	Group C	Group D
CTRL	I_2	GW9662	GW+I_2
0.832715530	0.519391980	0.767876200	0.766254550
1.210745500	0.618092920	1.018111550	0.809944880
0.956538960	0.695390680	0.973258970	0.753090760

Figure S4 Xenograft density and angiogenesis in zebrafish

Mean fluorescence intensity

Group A	Group B
Control	I_2
32443.8830000	58333.9400000
41952.0410000	33947.3840000
49684.4770000	35646.2380000
26714.1120000	36461.8840000
56553.6240000	84933.3400000
43760.1510000	76877.8530000
36652.2820000	28985.4320000
	24651.9940000

% Relative tumoral angiogenesis

Group A	Group B
Control	I_2
51.37440904	2.23678232
88.37865158	38.54814600
18.54907341	28.11080570
37.10187193	26.01077990
77.06859306	29.96432350
77.63416823	18.83814940
66.86817275	71.23961080
	43.08466250

Figure S5
Caudal migration of SK-N-BE(2) cells in zebrafish

Group A	Group B
Control	I_2
15	2
12	6
5	16
7	7
6	9
20	6
2	12
5	3
14	0
13	18
14	5
4	4
8	7
4	19
5	3
10	2
10	4
6	5
9	4
2	5
2	0
	3
	4
	0
	4
	0
	5
	2
	3
	4
	0
